# Sweet syndrome with reactive lymphadenopathy mimicking metastatic disease in an *SDHB*-mutated abdominal paraganglioma

**DOI:** 10.1210/jcemcr/luag135

**Published:** 2026-06-05

**Authors:** Leonardo V Carvalho, Aley Talans, Ibere C Soares, Marcelo Arnone, George B Coura-Filho, Madson Q Almeida

**Affiliations:** Unidade de Adrenal, Laboratório de Endocrinologia Molecular e Celular LIM25, Divisão de Endocrinologia e Metabologia, Hospital das Clínicas, Faculdade de Medicina da Universidade de São Paulo, São Paulo 01246-903, Brazil; Instituto de Radiologia (InRad), Hospital das Clínicas, Faculdade de Medicina da Universidade de São Paulo, São Paulo 01246-903, Brazil; Divisão de Anatomia Patológica, Instituto do Câncer do Estado de São Paulo (ICESP), Faculdade de Medicina da Universidade de São Paulo, São Paulo 01246-000, Brazil; Departamento de Dermatologia, Hospital das Clínicas, Faculdade de Medicina da Universidade de São Paulo, São Paulo 01246-903, Brazil; Medicina Nuclear, Instituto do Câncer do Estado de São Paulo (ICESP), Faculdade de Medicina da Universidade de São Paulo, São Paulo 01246-000, Brazil; Unidade de Adrenal, Laboratório de Endocrinologia Molecular e Celular LIM25, Divisão de Endocrinologia e Metabologia, Hospital das Clínicas, Faculdade de Medicina da Universidade de São Paulo, São Paulo 01246-903, Brazil; Unidade de Oncologia Endócrina, Instituto do Câncer do Estado de São Paulo (ICESP), Faculdade de Medicina da Universidade de São Paulo, São Paulo 01246-000, Brazil

**Keywords:** paraganglioma, sweet syndrome, *SDHB*, lymphadenopathy, paraneoplastic syndrome

## Abstract

We describe a case of Sweet syndrome, an acute neutrophilic dermatosis that may occur as a paraneoplastic manifestation. A 25-year-old man presented with recurrent abdominal pain and painful erythematous plaques on the trunk and upper limbs, without symptoms of catecholamine excess. Imaging revealed a retroperitoneal mass and extensive hypermetabolic lymphadenopathy on positron emission tomography/computed tomography using fluorine-18 fluorodeoxyglucose (^18^F-FDG) and gallium-68 DOTA–Tyr3–octreotate (^68^Ga-DOTATATE), raising concern for metastatic disease. Skin biopsy confirmed Sweet syndrome, while lymph node biopsies demonstrated reactive lymphoid hyperplasia. A computed tomography–guided biopsy of the retroperitoneal mass established the diagnosis of paraganglioma, but was complicated by hypertensive crisis. Subsequent biochemical evaluation revealed markedly elevated plasma normetanephrine levels despite normotension. The patient underwent preoperative alpha-adrenergic blockade followed by surgical resection. Cutaneous lesions resolved rapidly after tumor removal, and plasma normetanephrines normalized, confirming biochemical cure. This case highlights an association between Sweet syndrome and succinate dehydrogenase complex iron-sulfur subunit B *(SDHB)-*related paraganglioma and underscores the risk of false-positive metastatic findings on functional imaging and reinforces the importance of biochemical exclusion of paraganglioma before biopsy of vascularized retroperitoneal masses.

## Introduction

Sweet syndrome is an acute neutrophilic dermatosis characterized by the abrupt onset of fever, leukocytosis, and painful erythematous papules or plaques, histologically marked by a dense dermal infiltrate of mature neutrophils without evidence of vasculitis [1, 2]. The condition may occur idiopathically, be drug-induced, or arise as a paraneoplastic manifestation [1]. Although paraneoplastic Sweet syndrome is most commonly associated with acute myeloid leukemia and other hematologic malignancies, approximately 15% to 20% of cases are linked to solid tumors [3].

To date, only a few cases have reported an association between Sweet syndrome and catecholamine-secreting pheochromocytomas [4, 5]. Here, we report a case of Sweet syndrome associated with a succinate dehydrogenase complex iron-sulfur subunit B *(SDHB)*-mutated abdominal paraganglioma in a young adult, complicated by diffuse lymphadenopathy initially suggestive of metastatic disease.

## Case presentation

A previously healthy 25-year-old man presented with recurrent episodes of intense abdominal pain and persistent tender erythematous plaques involving the trunk and upper limbs, with low-grade fever and malaise. He denied recent infections, medication changes, or a family history of endocrine neoplasia or autoinflammatory disease. Notably, he did not report palpitations, episodic hypertension, diaphoresis, headaches, or other symptoms suggestive of catecholamine excess.

On physical examination, the patient was hemodynamically stable and normotensive. Dermatologic evaluation revealed multiple tender erythematous papules and plaques distributed over the anterior chest, upper back, and arms, without ulceration or mucosal involvement. Abdominal examination elicited discomfort on deep palpation of the left lower quadrant. Cardiopulmonary and neurologic examinations were otherwise unremarkable. Initial differential diagnoses included acute neutrophilic dermatosis, drug eruption, and infectious etiologies, prompting further laboratory and imaging evaluation. The patient had previously undergone a computed tomography (CT) scan at another institution, which revealed a retroperitoneal mass, and was subsequently referred to our center for further investigation.

## Diagnostic assessment

At initial hospital admission, laboratory evaluation revealed marked systemic inflammation, with elevated C-reactive protein levels of 103.5 mg/L (reference range, <5.0 mg/L), neutrophil-predominant leukocytosis, with an absolute neutrophil count of 7590 cells/mm^3^ (SI: 7.59 × 10^9^/L) (reference range, 1500-7500 cells/mm^3^; SI: 1.5-7.5 × 10^9^/L), and normocytic anemia with a hemoglobin level of 9.3 g/dL (SI: 93 g/L) (reference range, 13.5-17.5 g/dL; SI: 135-175 g/L).

Fluorine-18 fluorodeoxyglucose positron emission tomography/CT (^18^F-FDG PET/CT) demonstrated a heterogeneous hypermetabolic nodular lesion in the pancreatic head, measuring approximately 5.1 × 4.0 cm, with intense radiotracer uptake (maximum standardized uptake value [SUVmax], 28.2); anatomical characterization was limited due to the absence of iodinated contrast (Fig. 1). Additionally, multiple hypermetabolic lymph nodes were identified, including bilateral cervical lymph nodes at levels II–III (SUVmax 2.5, subcentimetric), thoracic lymphadenopathy involving the right upper paratracheal, subaortic, bilateral hilar, subcarinal, and bilateral axillary chains, as well as subcutaneous involvement of the left anterior chest wall (SUVmax up to 4.0, right axillary), measuring up to 2.4 cm. Abdominopelvic lymphadenopathy was also observed in the left external iliac and bilateral inguinal chains, with the largest node measuring up to 7.1 cm and demonstrating increased uptake (SUVmax 5.7, left inguinal).

**Figure 1:**
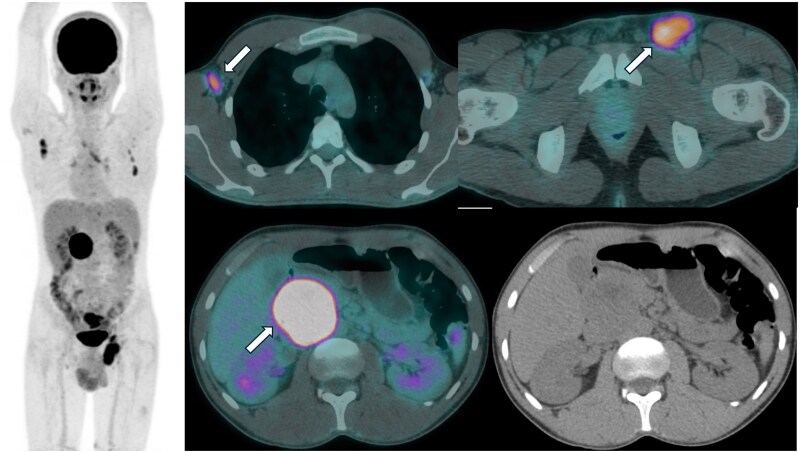
Fluorine-18 fluorodeoxyglucose positron emission tomography/computed tomography (^18^F-FDG PET/CT) demonstrates increased radiotracer uptake in a retroperitoneal mass located between the aorta and the inferior vena cava. Increased uptake is also observed in bilateral axillary lymph nodes, as well as in mediastinal, pulmonary hilar, left obturator, and inguinal lymph nodes. Areas of increased uptake are indicated by white arrows.

A skin biopsy revealed a dense neutrophilic infiltrate in the superficial dermis without evidence of vasculitis, consistent with Sweet syndrome (Fig. 2). To investigate a possible hematologic disorder in the context of a retroperitoneal mass associated with axillary and inguinal lymphadenopathy, excisional biopsies of axillary and inguinal lymph nodes were performed. Histopathologic and immunohistochemical analyses of both specimens were consistent with reactive lymphoid hyperplasia, with no evidence of malignancy. As both biopsies revealed no malignancy, a CT-guided biopsy of the retroperitoneal mass was subsequently performed. The immunohistochemical profile, characterized by diffuse chromogranin A positivity, S100 protein positivity in rare sustentacular cells, and absence of epithelial, hematolymphoid, gastrointestinal, and renal lineage markers, is consistent with a diagnosis of paraganglioma. During the procedure, the patient experienced a hypertensive crisis, with blood pressure peaking at 240/130 mmHg.

**Figure 2:**
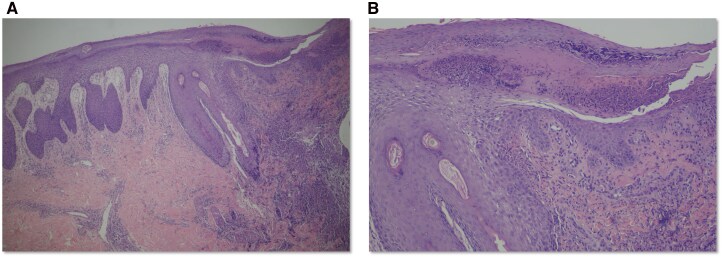
Skin histopathology consistent with sweet syndrome, showing crusts, hyperkeratosis, irregular acanthosis, spongiosis, and intraepidermal abscesses. The dermis exhibits a mixed neutrophil-rich inflammatory infiltrate, both interstitial and perifollicular. Hematoxylin and eosin staining; original magnification ×40 (A) and ×200 (B).

After referral to the endocrinology service for evaluation of paraganglioma, preoperative plasma normetanephrine levels were markedly elevated at 25.39 nmol/L (4.646 ng/mL; reference <0.90 nmol/L [<0.165 ng/mL]). Subsequent PET/CT using gallium-68 DOTA–Tyr^3^–octreotate (^68^Ga-DOTATATE) revealed persistent somatostatin receptor–avid uptake in the retroperitoneal mass and lymphadenopathy (Fig. 3). In contrast, iodine-123 metaiodobenzylguanidine (^123^I-MIBG) scintigraphy showed tracer uptake confined to the primary tumor, suggesting inflammatory rather than metastatic nodal involvement (Fig. 4).

**Figure 3:**
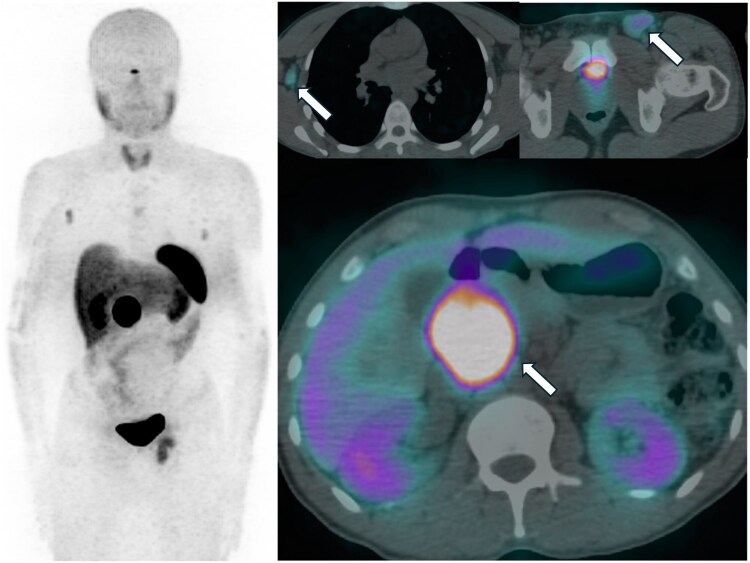
PET/CT using gallium-68 DOTA-Tyr^3^-octreotate (^68^Ga-DOTATATE) shows increased radiotracer uptake in the retroperitoneal mass located between the aorta and the inferior vena cava. Increased uptake is observed in bilateral axillary and left inguinal lymph nodes; however, no significant uptake is seen in mediastinal, pulmonary hilar, or left obturator lymph nodes. Areas of increased uptake are indicated by white arrows.

**Figure 4:**
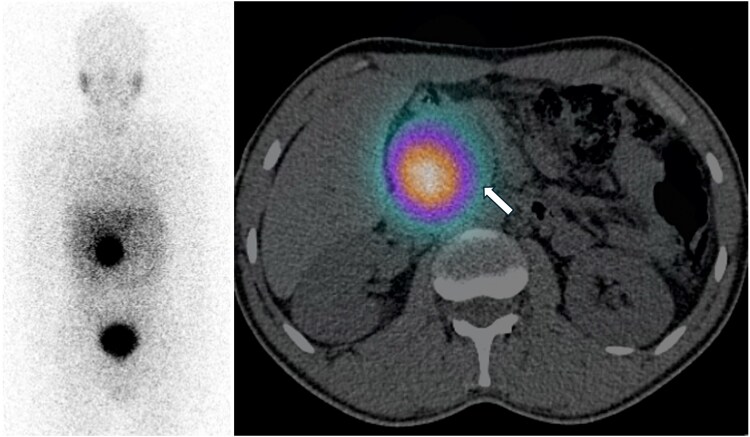
Iodine-123 metaiodobenzylguanidine (^123^I-MIBG) scintigraphy demonstrates increased radiotracer uptake in the retroperitoneal mass located between the aorta and the inferior vena cava. No abnormal uptake is observed in thoracic or pelvic lymph nodes. Area of increased uptake is indicated by white arrow.

## Treatment

Although the patient was normotensive, he underwent preoperative alpha-adrenergic blockade with doxazosin (1 mg twice daily), along with increased salt and fluid intake. Doxazosin treatment did not influence either cutaneous or nodal findings. During the diagnostic workup, topical corticosteroids were prescribed for the cutaneous lesions, resulting in partial improvement of skin manifestations but without any appreciable effect on the associated lymphadenopathy. No systemic corticosteroid therapy was initiated before tissue diagnosis because of concern for masking alternative diagnoses, including metastatic disease or infection, in the context of extensive hypermetabolic lymphadenopathy on functional imaging. After 30 days, the patient underwent complete surgical resection of the retroperitoneal paraganglioma. Histopathologic examination revealed a paraganglioma with a classic Zellballen growth pattern, strong chromogranin A and synaptophysin expression, and S100-positive sustentacular cells. No tumor necrosis, vascular, or perineural invasion was identified, and the Ki-67 (marker of cellular proliferation) index was approximately 1%. Tumor grading revealed a Pheochromocytoma of the Adrenal Gland Scaled Score (PASS) of 5, a Grading System for Adrenal Pheochromocytoma and Paraganglioma (GAPP) score of 6, and a modified GAPP (M-GAPP) score of 5, consistent with a moderately differentiated neuroendocrine neoplasm.

## Outcome and follow-up

Following tumor resection, cutaneous lesions resolved spontaneously within two weeks, without the need for immunosuppressive therapy, and plasma normetanephrine levels normalized to 0.30 nmol/L (0.055 ng/mL), confirming biochemical cure. Over more than two years of follow-up, the patient remained clinically and biochemically stable.

Genetic investigation identified a heterozygous germline deletion of exon 1 in the *SDHB* gene. In light of this germline *SDHB* pathogenic variant, long-term surveillance was initiated in accordance with current guidelines for hereditary *SDHB*-related paraganglioma syndromes [6]. Head CT and abdominal magnetic resonance imaging (MRI) were unremarkable after two years of follow-up.

## Discussion

Sweet syndrome, originally described by Sweet in 1964, is characterized by the abrupt onset of painful erythematous skin lesions, fever, and a dense dermal neutrophilic infiltrate without evidence of vasculitis [7, 8]. Current evidence suggests that tumor-related immune dysregulation plays a central role, with increased production of proinflammatory cytokines such as interleukin-1 (IL-1), interleukin-6 (IL-6), tumor necrosis factor-α (TNF-α), and granulocyte colony-stimulating factor (G-CSF), leading to neutrophil activation, recruitment, and tissue infiltration [9, 10]. Elevated circulating levels of G-CSF and IL-6 have been reported in several cases of paraneoplastic Sweet syndrome, supporting a cytokine-mediated mechanism [10]. These cytokines are considered key molecular mediators of the paraneoplastic inflammatory response. In catecholamine-secreting tumors, additional immunomodulatory effects of catecholamines and tumor-driven inflammatory signaling may further contribute to systemic neutrophilic inflammation [11]. Although specific circulating mediators were not measured, the proposed cytokine-driven mechanisms provide a biologically plausible explanation for a paraneoplastic association.

In the present case, Sweet syndrome was associated with an *SDHB*-mutated abdominal paraganglioma, an association that has rarely been reported in the literature and suggests a possible link between this genotype and paraneoplastic neutrophilic dermatosis. The presence of extensive lymphadenopathy with intense uptake on ^18^F-fluorodeoxyglucose and ^68^Ga-DOTATATE PET/CT closely mimicked metastatic disease, posing a major diagnostic challenge. Although the sensitivity of ^123^I-metaiodobenzylguanidine scintigraphy for detecting *SDHB*-related paragangliomas and metastatic disease is lower than that of ^68^Ga-DOTATATE PET/CT [12, 13], histopathological analysis demonstrated reactive lymphoid hyperplasia rather than metastatic involvement, indicating that lymphadenopathy and Sweet syndrome represented concurrent but pathologically distinct inflammatory manifestations. Plasma metanephrine levels normalized after paraganglioma resection, underscoring that inflammatory paraneoplastic phenomena may lead to false-positive findings on functional imaging, except for MIBG, in patients with paraganglioma. Notably, increased ^18^F-fluorodeoxyglucose uptake is commonly observed in these tumors, particularly in those harboring *SDHB* pathogenic variants, even when the Ki-67 proliferative index is low. In this context, FDG avidity reflects tumor metabolic reprogramming and pseudohypoxic signaling rather than proliferative activity per se. Therefore, intense ^18^F-fluorodeoxyglucose uptake should not be interpreted as a surrogate marker of aggressive histology or high proliferative index in *SDHB*-related paragangliomas [12].

A limited number of cases have reported an association between Sweet syndrome and catecholamine-secreting pheochromocytomas, typically in patients without known hereditary pathogenic variants and without extensive lymphadenopathy [4, 5]. In contrast, our patient harbored a germline *SDHB* pathogenic variant, a genotype strongly associated with extra-adrenal location and increased metastatic risk. This clinical context heightened concern for disseminated disease and underscores the importance of careful interpretation of imaging findings in *SDHB*-related paragangliomas, where inflammatory or immune-mediated reactions may coexist with or masquerade as metastatic lesions.

This case also emphasizes a critical and well-established principle in diagnostic assessment that paraganglioma must be biochemically excluded before biopsy of a vascularized retroperitoneal mass [14, 15]. Measurement of plasma or urinary metanephrines is essential before any invasive diagnostic procedure. Failure to recognize a catecholamine-secreting tumor can result in life-threatening complications, as illustrated by the hypertensive crisis experienced by our patient during CT-guided biopsy. Despite the absence of classic symptoms of catecholamine excess and normotension at presentation, markedly elevated plasma normetanephrine levels were subsequently identified, reinforcing that biochemical screening should not be omitted based on clinical presentation alone [16].

Although our patient was normotensive at presentation, plasma normetanephrine levels were markedly elevated, indicating significant catecholamine excess and potential hemodynamic instability during surgical manipulation. This underscores the importance of preoperative alpha-adrenergic blockade in patients with biochemically active paragangliomas, even in the absence of sustained hypertension [17]. Current guidelines recommend alpha-adrenergic blockade for all patients with catecholamine-secreting pheochromocytomas and paragangliomas to reduce the risk of perioperative cardiovascular complications, regardless of baseline blood pressure [14, 15]. The use of doxazosin, combined with adequate salt and fluid intake, was well tolerated in this patient and allowed safe surgical resection, reinforcing that normotension does not exclude the need for appropriate preoperative pharmacologic preparation.

Patients with *SDHB*-mutated paragangliomas require lifelong surveillance owing to the substantial risk of local recurrence, metachronous tumors, and metastatic disease associated with this genotype. In the present case, genetic investigation identified a heterozygous germline deletion of exon 1 in the *SDHB* gene. Consistent with current international consensus recommendations, long-term follow-up was instituted, including periodic biochemical assessment and whole-body imaging to allow early detection of recurrence or metastatic spread [6]. After complete surgical resection, follow-up imaging with head CT and abdominal MRI remained unremarkable over more than two years, and plasma normetanephrine levels stayed within the normal range, supporting sustained disease remission while underscoring the need for continued lifelong surveillance in this high-risk population.

The treatment of Sweet syndrome primarily consists of systemic corticosteroids, but it also depends on the underlying cause (idiopathic, malignancy-associated, or drug-induced). Given that our patient underwent complete tumor resection with subsequent improvement of cutaneous manifestations and stable lymphadenopathy (without regression), we did not initiate systemic corticosteroid therapy. The complete resolution of cutaneous lesions and normalization of plasma normetanephrines after tumor resection support a paraneoplastic mechanism and emphasize the importance of multidisciplinary evaluation to avoid misdiagnosis.

## Learning points

- Sweet syndrome may be associated with catecholamine-secreting paragangliomas, including *SDHB*-mutated tumors, and may mimic metastatic disease on functional imaging.
- Extensive lymphadenopathy with increased uptake on ^18^F-FDG and ^68^Ga-DOTATATE PET/CT does not necessarily indicate metastatic involvement in paraganglioma and may reflect inflammatory or immune-mediated reactions.
- Paraganglioma must be biochemically excluded with plasma or urinary metanephrine measurements before biopsy of a vascularized retroperitoneal or adrenal mass to avoid potentially life-threatening complications.
- Normotension does not exclude clinically significant catecholamine excess, and preoperative alpha-adrenergic blockade is indicated in biochemically active paragangliomas regardless of baseline blood pressure.
- Although systemic corticosteroids are the mainstay of treatment for Sweet syndrome, careful evaluation of the underlying cause (idiopathic, malignancy-associated, or drug-induced) is essential before initiation to avoid masking alternative diagnoses.

## Data Availability

Data sharing is not applicable to this article as no datasets were generated or analyzed during the current study.
